# Supplementary material for: The critical role of Hedgehog-responsive mesenchymal progenitors in meniscus development and injury repair
Source: eLife. 2021 Jun 4;10:e62917. doi: 10.7554/eLife.62917 (PMC8177886; doi:10.7554/eLife.62917)
Supplement: Supplementary file 1. [file elife-62917-supp1.docx]

**Table S1. Mouse real-time PCR primer sequences.**

| Gene | Forward primer | Reverse primer |
| --- | --- | --- |
| *Gli1* | 5’-CCACCCTACCTCTGTCTATT-3’ | 5’-CCATTGCCCATCACAGAA-3’ |
| *Gli2* | 5’-GTTCCAAGGCCTACTCTCGCCTG-3’ | 5’-CTTGAGCAGTGGAGCACGGACAT-3’ |
| *Gli3* | 5’-AGCAACCAGGAGCCTGAAGTCAT-3’ | 5’-GTCTTGAGTAGGCTTTTGTGCAA-3’ |
| *Ihh* | 5’-CAGACCGTGACCGAAATAAG-3’ | 5’-GCCTTGGACTCGTAATACAC-3’ |
| *Dhh* | 5’-TCTGACCGTGACCGTAAT-3’ | 5’-CTTTGACCGATACGTGGATG-3’ |
| *Shh* | 5’-CCATCTCTGTGATGAACCAG-3’ | 5’-CGACCCTCATAGTGTAGAGA-3’ |
| *Smo* | 5’-GAGCGTAGCTTCCGGGACTA-3’ | 5’-CTGGGCCGATTCTTGATCTCA-3’ |
| *Ptch1* | 5’-AAAGAACTGCGGCAAGTTTTTG-3’ | 5’-CTTCTCCTATCTTCTGACGGGT-3’ |
| *Hhip* | 5’-TGAAGATGCTCTCGTTTAAGCTG-3’ | 5’-CCACCACACAGGATCTCTCC-3’ |
| *Pparg* | 5’-GCCCTTTGGTGACTTTATGGA-3’ | 5’-GCAGCAGGTTGTCTTGGATG-3’ |
| *Lpl* | 5’-GCGCTCCATCCATCTCTTCAT-3’ | 5’-GGCAGAGCCCTTTCTCAAATG-3’ |
| *Ibsp* | 5’-GGCCACGCTACTTTCTTT-3’ | 5’-CCCTCCTCTTCGGAACTAT-3’ |
| *Osx* | 5’-ACTCATCCCTATGGCTCGTG-3’ | 5’-GGTAGGGAGCTGGGTTAAGG-3’ |
| *β-actin* | 5’-TCCTCCTGAGCGCAAGTACTCT-3’ | 5’-CGGACTCATCGTACTCCTGCTT-3’ |
| *Col2a1* | 5’-CATTGCCTATCTGGACGAAG-3’ | 5’-GTGTACGTGAACCTGCTATT-3’ |
| *Sox9* | 5’-AGGAGAGCGAGGAAGATAAG-3’ | 5’-ACGTGTGGCTTGTTCTTG-3’ |
